# Effect of HO-3089 PARP inhibitor on inflammatory response

**DOI:** 10.1186/cc9672

**Published:** 2011-03-11

**Authors:** M Nemeth, T Leiner, K Tanczos, A Mikor, Z Molnar, K Kovacs

**Affiliations:** 1 University of Pecs, Hungary; 2 University of Szeged, Hungary

## Introduction

The activation of poly-ADP-ribose-polymerase enzyme (PARP) plays an important role in the pathophysiology of sepsis [1]. In previous animal models, lipopolysaccharide-induced systemic inflammatory response was significantly reduced by the inhibition of PARP [2]. The aim of our study was to investigate the effect of PARP inhibition on systemic inflammation in a septic animal model.

## Methods

In a prospective, randomized study, anaesthetized CFY rats were divided into four groups (five/group): cecal ligation group (CL), cecal ligation and punction group (CLP), CLP with PARP inhibition (CLP+Pi) group and sham group. PARP inhibition was performed by HO-3089 (a novel PARP inhibitor) given intraperitoneally (10 mg/kg). Heart rate, invasive blood pressure and the rectal temperature were monitored. Data were recorded every 15 minutes. To identify the inflammatory response, IL-6 and TNFα were measured by quantitative sandwich enzyme immunoassay technique. Blood samples were taken before the CLP (t_0_), 2 hours (t_1_) and 6 hours (t_2_) after the CLP.

## Results

IL-6 and TNFα were significantly higher in the CLP and CLP+Pi groups at t_2 _as compared with t_0 _(CLP: *P*_IL-6 _< 0.001, *P*_TNFα _< 0.001; CLP+Pi:*P*_IL-6 _= 0.002, *P*_TNFα _< 0.001), and also as compared with the CL and sham groups at t_2 _(CL vs. CLP: *P*_IL-6 _< 0.001, *P*_TNFα _= 0.002; CL vs. CLP+Pi: *P*_IL-6 _= 0.008, *P*_TNFα _= 0.002; sham vs. CLP: p_IL-6 _< 0.001, *P*_TNFα _= 0.002; sham vs. CLP+Pi: *P*_IL-6 _= 0.011, *P*_TNFα _= 0.002). Although in the CLP+Pi group theIL-6 level was lower than in the CLP group at t_2_, but the difference was not significant (*P *= 0.074). There was no significant difference in TNFα between the CLP and CLP+Pi groups either.

## Conclusions

The initial results of this study could not show a significant effect of the HO-3089 PARP inhibitor in CLP caused systemic inflammatory response. However, the tendency of lower IL-6 in the treated group warrants the completion of the experiment.
